# Dietary Diversity During Complementary Feeding and Its Association with Nutritional Status Among Infants and Young Children in Indonesia: A Literature Review

**DOI:** 10.3390/children13101400

**Published:** 2026-10-14

**Authors:** Ray Wagiu Basrowi, Nova Lidia Sitorus, Dessy Pratiwi, Addini Pascaramadhani

**Affiliations:** 1 Occupational Medicine Division, Department of Community Medicine, Faculty of Medicine, Universitas Indonesia, Jakarta 10320, Indonesia; ray.basrowi@gmail.com; 2 Health Collaborative Center, TB Simatupang 18C, Jakarta 12430, Indonesia; nova.lidia.sitorus@gmail.com; 3 Indonesia Health Development Center, East Kuningan, Jakarta 12950, Indonesia; pratiwi.dessy@gmail.com; 4 Nutrition Program, Faculty of Health Sciences, Yarsi Pratama University, Tangerang 15720, Indonesia

**Keywords:** complementary feeding, dietary diversity, nutritional status, stunting, Indonesia

## Abstract

**Highlights:**

**What are the main findings?**

- Higher dietary diversity during complementary feeding was generally associated with more favorable anthropometric outcomes, particularly linear growth and stunting, although associations varied across settings and were not consistently independent after adjustment.
- In Indonesia, dietary diversity was associated with socioeconomic, maternal, cultural, and food-access factors, indicating that complementary feeding practices are shaped by broader household and food-system contexts.

**What are the implications of the main findings?**

- Complementary feeding support should consider context-specific nutrition education and behavior-change strategies that address caregiver practices, affordability, accessibility, and cultural barriers, using existing Posyandu and Puskesmas platforms.
- Dietary diversity may be integrated as one component of broader child nutrition strategies, including interprofessional health services and food-based nutrition programs, while the effectiveness of specific implementation approaches requires further evaluation.

**Abstract:**

Dietary diversity is an important component of complementary feeding and may influence the nutritional status and growth of infants and young children. This review aimed to synthesize evidence on the association between dietary diversity during complementary feeding and nutritional status, with emphasis on Indonesia and relevant international evidence. This study was conducted as a targeted narrative review. A targeted literature search was conducted using PubMed/MEDLINE and Google Scholar between January and March 2026. Studies were eligible if they included children aged 6–23 months, assessed dietary diversity or minimum dietary diversity, reported nutritional outcomes, and were original research articles published in English between 2004 and 2026. Findings were synthesized narratively because of heterogeneity in study designs, populations, dietary diversity measures, and nutritional outcomes. Sixteen studies met the eligibility criteria, comprising 10 Indonesian and 6 non-Indonesian or multi-country studies. Overall, higher dietary diversity was generally associated with more favorable anthropometric outcomes, particularly linear growth and lower odds of stunting, although associations varied across settings and after adjustment for other factors. In Indonesia, socioeconomic, maternal, cultural, and food-access factors were also associated with dietary diversity. Dietary diversity may be associated with more favorable nutritional and growth outcomes among children aged 6–23 months. Improving dietary diversity in Indonesia may benefit from context-specific approaches addressing caregiver practices, food-system constraints, and community and primary healthcare capacity.

## 1. Introduction

The first 1000 days, spanning from conception to approximately two years of age, represent a critical window for growth and development. Poor infant nutritional status, as well as maternal nutrition and health during this period, have been associated with impaired linear growth, cognitive and physical development, immune function, and longer-term health outcomes [1]. In Indonesia, the prevalence of infant and preschool children affected by undernutrition continues to be an important concern in public health, despite continuing efforts to enhance the nutritional status of mothers and children. It has also been revealed in the Indonesian Basic Health Research Survey, or Riskesdas 2018, that the prevalence of stunting in young children was still high at 30.8%, pointing to the susceptibility of the age group to poor feeding habits [2].

Exclusive breastfeeding is recommended for the first six months of life, but human milk alone cannot meet the growing energy and micronutrient needs of infants aged more than six months [3]. Appropriate complementary feeding is therefore necessary to further support growth and development. The habitual pattern of complementary feeding in Indonesia often consists of the early introduction of rice-based foods such as plain porridge or mashed rice, combined with a limited dietary diversity characterized by low animal-source foods, fruits, and vegetable intake [4]. Diets dominated by such complementary foods have been shown to be inadequate in several essential micronutrients, including iron, zinc, and calcium, which are critical for optimal child growth and immune function [5].

Dietary diversity has been used as a proxy indicator of diet quality and micronutrient adequacy in infant and young child feeding practices. The World Health Organization defines minimum dietary diversity as the consumption of food from at least five out of eight recommended food groups within a 24 h period among children aged 6–23 months [6]. Earlier studies included in this review used the previous minimum dietary diversity definition of consuming at least four of seven food groups. This indicator was subsequently updated to five of eight food groups following the 2017 expert consultation, with breast milk included as the eighth food group [6].

In the Indonesian context, national and subnational studies have shown that a substantial proportion of children fail to meet minimum dietary diversity, with low dietary diversity identified as a component of inadequate complementary feeding practices linked to child stunting [7]. Additionally, socioeconomic and geographical disparities can add to the challenge of suboptimal child-feeding practices in Indonesia. Evidence indicates that children from impoverished environments and rural areas are most adversely impacted by the lack of services for proper nutrition, as well as information regarding that very same nutrition [7,8]. The education level of mothers has long been identified as playing a significant role in determining the quality of complementary feeding [7,9]. Moreover, low education has been associated with a lack of diversity and delays in children’s exposure to nutrient-rich diets [9].

With Indonesia’s persistent efforts in preventing stunting through national programs aiming at the first 1000 days of life, promoting dietary diversity during the complementary feeding phase remains one of the key priorities [1,7]. It has become vital to comprehend the link between dietary diversity and nutritional status in Indonesian infants and young children in order to make informed policies and further strengthen nutrition improvement programs in Indonesia [7].

Although evidence from global and regional studies is also considered, findings applicable to the Indonesian setting are a priority. At the same time, the international literature is drawn upon for conceptual, biological, and methodological perspectives. Even with a vast amount of global literature in existence, considerable knowledge gaps remain for the Indonesian population. Firstly, most of the existing reviews have focused mainly on dietary diversity as a single composite indicator rather than examining potential biological pathways that may help explain its relationship with child growth, such as deficiencies in specific micronutrients, imbalances in the microbiota, and hydration levels [10,11,12]. Another issue is that even though the nutritional requirements of children aged 6–23 months have been well established, there is limited information about the relationship between dietary diversity and meeting specific nutrient composition targets, especially those associated with animal-source foods [5,13].

Moreover, although national strategies and programs have emphasized the strengthening of nutrition services and interventions in Indonesia [14,15], limited evidence is available regarding how these strategies can be optimized to support complementary feeding practices among infants and young children. These gaps need to be addressed to develop evidence-based recommendations that may help speed up efforts at reducing the prevalence of stunting in Indonesia. Thus, this review aims to provide a synthesis of the evidence on the link between dietary diversity and nutrition in infant and young child feeding, with emphasis on research conducted in Indonesia and other countries, as well as factors that may explain these associations.

## 2. Materials and Methods

### 2.1. Literature Search

This study was conducted as a targeted narrative review to synthesize available evidence on dietary diversity during complementary feeding and its association with nutritional status among children aged 6–23 months, with particular attention to the Indonesian context, while relevant international studies were also considered to provide comparative and contextual evidence. A targeted literature search was conducted using major electronic databases, including PubMed/MEDLINE and Google Scholar from 28 January to 20 March 2026. The search used combinations of the following keywords: “dietary diversity”, “minimum dietary diversity”, “complementary feeding”, “infant and young child feeding”, “nutritional status”, “stunting”, “undernutrition”. These keywords were combined using Boolean operators (AND/OR), with the documented search strategy as follows: (“dietary diversity” OR “minimum dietary diversity”) AND (“complementary feeding” OR “infant and young child feeding”) AND (“nutritional status” OR stunting OR undernutrition) AND (Indonesia OR “low-and middle-income countries”). The age criterion of 6–23 months was applied during the screening process.

### 2.2. Eligibility Criteria

Studies were considered eligible when they met the following criteria: (1) included children aged 6–23 months; (2) assessed dietary diversity, minimum dietary diversity, or complementary feeding where dietary diversity was an exposure- or outcome-related variable; (3) reported nutritional status, growth or anthropometric outcomes; and (4) were original research studies; and (5) were published in English between 2004 and 2026. The age range of 6–23 months was selected to align with the age range specified for the minimum dietary diversity indicator in the WHO/UNICEF infant and young child feeding framework [6]. Reviews and other supporting publications were retained when they provided relevant contextual, biological, methodological, or conceptual information for interpretation of the reviewed evidence.

### 2.3. Study Selection and Data Synthesis

Articles were screened for relevance based on their titles, abstracts, and, where necessary, full-texts, and were subsequently assessed against the aforementioned inclusion criteria. Relevance was defined as direct alignment with the review objectives, particularly whether the study examined dietary diversity or related complementary feeding practices among children aged 6–23 months and reported nutritional or growth-related outcomes. The selected studies were reviewed for study design, population characteristics, dietary diversity or complementary feeding measures, nutritional outcomes, and key findings. The data from these articles were extracted and summarized descriptively in Table 1. Studies that did not address the review population, exposure, or relevant nutritional outcomes were not included in the evidence synthesis. As different types of studies were included in this review, quantitative synthesis was not performed due to different study designs, population characteristics, dietary diversity measures, and nutritional outcomes across the included studies. Instead, the findings were synthesized narratively, considering the association between dietary diversity during complementary feeding and nutritional status, as well as relevant contextual factors.

## 3. Results

A total of 15 research studies met the predefined eligibility criteria and were included in the primary evidence synthesis. The characteristics of the included studies are summarized in Table 1. The included studies were distributed between Indonesian settings (*n* = 10) and non-Indonesian or multi-country settings (*n* = 5). The included studies employed diverse methodological approaches, including cross-sectional studies, prospective cohort research, and secondary analyses of nationally representative survey data. All studies focused on infants and young children, primarily within the age range of 6–23 months.

Dietary diversity was operationalized using various measures including minimum dietary diversity (MDD), dietary diversity score (DDS), and WHO/UNICEF IYCF indicators. Nutritional outcomes assessed included weight-for-age z-score (WAZ), length-for-age z-score (LAZ), stunting, wasting, underweight, and other indicators of nutritional status. Some studies also examined specific food groups, particularly animal-source food intake, to provide further insight into dietary quality and its relationship with children’s nutritional outcomes.

## 4. Discussion

### 4.1. Dietary Diversity and Nutritional Status During Complementary Feeding

This literature review highlights the available evidence on the role of dietary diversity during the complementary feeding period as an important nutritional variable associated with nutritional status and growth in infants and young children. Dietary diversity provides an indicator of the variety of foods consumed during complementary feeding period and may serve as a proxy for aspects of diet quality and micronutrient adequacy [13,16].

Based on the evidence available in this literature review, the findings generally suggest that inadequate dietary diversity during the complementary feeding period is associated with poorer nutritional status and growth, including linear growth and stunting, although the strength and consistency of the association vary across populations and study designs. Multi-country analyses have found positive associations between dietary diversity and attained linear growth, while several studies in Indonesia have noted that inadequate dietary diversity is related to stunting and poor nutritional status [16,17,20,21,24,27,28,29]. Similarly, other multi-country analyses found that adequate MDD was associated with lower odds of stunting, underweight, and wasting [16,23].

Several studies from Indonesia also reported associations between dietary diversity and stunting. In Bantul, poor dietary diversity was strongly associated with stunting in multivariable analysis (OR = 16.76, 95% CI: 6.77–41.51) [17]. In another Indonesian study, poor dietary diversity was associated with higher odds of stunting after multivariable adjustment (OR = 2.91, 95% CI: 1.21–7.02) [21]. National Indonesian data from Riskesdas 2018 also showed an association between meeting MDD and nutritional status after adjustment, although the outcome was modeled as normal nutritional status rather than stunting directly [24]. More recent Indonesian analyses continued to report associations between inadequate MDD and stunting [27,29].

However, this association was not observed in some settings. In Aceh, no significant association between minimum dietary diversity and stunting was observed in the bivariate analysis [19]. In Northern Ghana, dietary diversity was not an independent predictor of stunting after adjustment for other factors such as child characteristics, household characteristics, and feeding practices [22]. These findings indicate that dietary diversity should not be considered an isolated determinant of nutritional status, as its observed association with child growth may also be influenced by socioeconomic conditions, child characteristics, illness, and other complementary feeding practices.

### 4.2. Dietary Diversity and Anthropometric Outcomes

Across multiple observational and secondary analyses, higher dietary diversity during the complementary feeding period has generally been associated with more favorable anthropometric outcomes. Children who met the minimum dietary diversity criteria generally showed more favorable anthropometric outcomes, including higher length-for-age measures, and lower odds of stunting, underweight, or wasting in several settings [16,23,24]. However, the strength and consistency of these associations varied across anthropometric outcomes and study settings.

Several studies reported associations between dietary diversity or MDD and indicators of linear growth. Onyango et al. identified positive associations between MDD and LAZ in several countries, although the magnitude and statistical significance of the associations varied across settings [16]. In contrast, a prospective study from West Java found no significant association between MDD and LAZ, WLZ, or WAZ at 12 months after adjustment [18].

Evidence was also observed for weight-related outcomes. Aboagye et al. reported that adequate MDD was associated with lower odds of stunting (aOR = 0.88, 95% CI: 0.83–0.94), underweight (aOR = 0.83, 95% CI: 0.77–0.91), and wasting (aOR = 0.87, 95% CI: 0.78–0.97) across 32 countries in sub-Saharan Africa [23]. In Vietnam, adequate MDD was associated with lower odds of stunting (aOR = 0.61, 95% CI: 0.41–0.92) [26]. However, not all studies demonstrated significant associations after adjustment. For example, the study from Northern Ghana did not identify MDD as an independent predictor of stunting or wasting [22], while the Tanzanian study also found no significant adjusted association between MDD and stunting, wasting, or underweight [30].

Overall, the reviewed evidence suggests that associations between dietary diversity and linear growth or stunting were observed across several settings, whereas findings for weight-related outcomes were more variable. Differences in study populations, dietary diversity definitions, dietary assessment methods, anthropometric indicators, and adjustment variables may contribute to the variation in observed associations.

### 4.3. Dietary Diversity, Nutrient Composition, and Potential Biological Pathways

To meet the criteria for a “balanced diet,” complementary diets should not only meet the minimum number of food groups but also provide adequate macronutrient and micronutrient composition. As far as macronutrients are concerned, the energy contribution required from complementary foods is approximately 200–550 kcal/day, depending on the age of the child, and should be provided together with adequate amounts of high-quality protein [13]. Animal source foods (ASFs), namely meat, eggs, fish, and milk, have an important place here as their proteins are complete and include all necessary amino acids, including lysine, leucine, and threonine that play a crucial role in muscle mass formation, tissue regeneration, and body growth [5,13,31]. On the contrary, plant protein sources in staple-dominant diets may have lower protein quality and digestibility and may provide limited amounts of some essential amino acids, such as lysine and methionine, when consumed in a monotonous diet [5,13]. In addition, a sufficient amount of fats (30–45% of total energy) is needed for vitamin A, D, E, and K absorption and for the purpose of nervous system myelination within the first two years of age [13].

Among micronutrients, some that need to be paid special attention to include iron, zinc, calcium, vitamin A, and vitamin B12. These can be provided by the use of ASF most effectively [5,13,18,32]. Iron is needed for hemoglobin production, transport of oxygen, and proper development of the brain. Heme iron, which is found in meat and fish, is absorbed by our body at a rate of 15–35%, while non-heme iron contained in plant food has only 2–8% absorbability [5,18]. Zinc is important for cell division, the immune system, and normal growth, and a lack of it contributes to stunted growth and increased risk of infections [5]. Calcium, working together with vitamin D, is crucial for mineralization of bone matrix and formation of bone; their deficiency results in stunted growth and rickets [13]. Vitamin A is responsible for maintaining epithelial barriers, immune functions, and vision, and its deficiency, which is rather common in Indonesia, increases the morbidity and stunting of children [7]. Vitamin B12 plays a role in DNA synthesis, red blood cell production, and neurogenesis, and its deficiency in infants on a mostly plant-based diet causes megaloblastic anemia and delayed development [13]. Magnesium is also an essential mineral involved in energy metabolism and bone development and may therefore be relevant to growth during childhood [33].

These nutrients provide plausible pathways through which dietary quality may be related to child growth, although the included studies did not directly establish micronutrient adequacy as a mediator of the association between dietary diversity and anthropometric outcomes. In the context of the particular micronutrient pathways, iron deficiency anemia may contribute to poor growth and may partly help explain the association observed between inadequate dietary diversity and stunting. Specifically, a systematic review and meta-analysis of pediatric populations in developing countries have shown that iron deficiency anemia significantly increases the risk of stunting, supporting the importance of adequate dietary iron intake during complementary feeding [10]. Zinc deficiency may impair normal growth and contribute to linear growth faltering [5,7]. Calcium, together with vitamin D, is crucial for bone mineralization and formation, and their deficiency may impair bone development and contribute to growth abnormalities [13]. Vitamin A deficiency has been associated with impaired growth and immune function, both of which may contribute to poor linear growth [7]. Collectively, these nutritional pathways provide a biologically plausible explanation for how inadequate dietary quality could contribute to impaired growth, but they should be interpreted as potential mechanisms rather than established mediating pathways in the studies included in this review.

Beyond micronutrient adequacy, the gut microbiome has also been proposed as a potential biological pathway linking dietary patterns and child growth. According to a scoping review of the literature on undernutrition and stunting in young children, alterations in the gut microbiome have been reported to be associated with undernutrition and stunting and may be linked to impaired nutrient utilization and linear growth [11]. However, the reviewed studies did not directly assess whether changes in the gut microbiome mediated the relationship between dietary diversity and nutritional status. Therefore, this pathway should be considered a plausible biological explanation rather than a demonstrated mechanism of the association observed in the present review.

Hydration may represent another contextual factor relevant to child nutritional status. A recent review of the literature on hydration has indicated that insufficient hydration in children under five is associated with poorer growth outcomes [12]. Nevertheless, hydration was not assessed as a mediator of the dietary diversity–growth relationship in the studies included in this review. Its relevance should therefore be interpreted as part of the broader nutritional and health context rather than as a demonstrated pathway linking dietary diversity to stunting.

### 4.4. Determinants of Low Dietary Diversity in Indonesia

Despite the documented association between dietary diversity and child nutritional outcomes, substantial variability in dietary diversity persists across low- and middle-income countries. Evidence from the literature suggests that socioeconomic conditions, maternal education, and access to nutrient-dense foods are associated with complementary feeding practices and dietary adequacy [4,5,7,9]. In Indonesia, higher household wealth and maternal education were associated with a greater likelihood of meeting minimum dietary diversity, while children from poorer households and those with less-educated mothers were more likely to experience inadequate dietary diversity [9].

Within the Indonesian context specifically, maternal nutrition literacy and nutrition knowledge have emerged as important potentially modifiable factors related to child nutritional outcomes. According to a recent scoping review of relevant evidence, maternal nutrition literacy and nutrition knowledge were generally associated with reduced stunting prevalence rates, suggesting that caregiver education may represent a potentially modifiable area for improving child nutrition [34]. Beyond maternal knowledge, sociodemographic and parenting practices are also associated with child feeding and nutritional outcomes. Studies proposed that factors related to complementary feeding practices include maternal and household characteristics, while parenting practices may also play a role in the relationship between household socioeconomic position and child stunting [9,35].

Importantly, these socioeconomic and maternal factors should be considered as contextual factors that may influence dietary diversity and nutritional outcomes, rather than as independent causal explanations. For example, differences in household economic status have been reported to modify or confound the association between dietary diversity and stunting in Indonesian children [9]. Similarly, some studies included in this review adjusted for socioeconomic and household-related factors when examining the association between dietary diversity and nutritional status, indicating the importance of considering these variables when interpreting the observed associations.

The relevance of food diversity is further underscored by the predominance of staple foods in complementary feeding. In the study by Paramita et al. [36], grains, roots, and tubers were consumed by more than 93% of children aged 6–23 months across the provinces of Java, while the prevalence of dietary diversity varied substantially between provinces. Although staple-based complementary foods play an important role in meeting energy requirements, they may provide relatively limited amounts of bioavailable iron, zinc, and high-quality protein. Evidence suggests that diets predominantly based on staple foods may provide limited amounts of some nutrients needed to support skeletal growth, immune function, and neurodevelopment during early childhood [5,13]. A study by Rah et al. reported that low dietary diversity was associated with a higher risk of stunting among children in rural Bangladesh [37]. In Indonesia, inadequate dietary diversity has also been reported among children aged 6–23 months, with socioeconomic and geographic factors contributing to differences in complementary feeding practices [9].

Animal-source foods are widely recognized as a key component of dietary diversification [38], and may make an important contribution to child growth and nutritional adequacy [39]. Evidence synthesized from intervention and observational studies suggests that inclusion of animal-source foods, such as eggs, fish, or meat, during complementary feeding may support linear growth and may be associated with a lower risk of stunting in young children [5,31,32,39]. This is particularly relevant given that relatively affordable animal source foods such as eggs and small fish appear to be widely available in Indonesia but remain underutilized as part of complementary feeding practices [40]. Linear programming analyses show that eggs, chicken liver, tempeh-tofu, and small fish can contribute to improving dietary quality during complementary feeding in Indonesia, further highlighting the gap between food availability and actual feeding practices [41]. Findings from recent observational studies suggest that caregivers’ beliefs regarding the health effects and digestibility of animal source foods, together with food availability and accessibility, may play an important role in shaping animal-source food consumption during complementary feeding [9,40]. These findings suggest that the contribution of animal-source foods to dietary diversity may depend not only on their nutritional value but also on household access, affordability, and caregiver practices. All of these issues highlight the relevance of dietary diversity as an intervention target in complementary feeding practices, as well as the need for behavior change interventions that are sensitive to local context.

The relationship between dietary diversity and nutritional status in Indonesia is also shaped by disparities based on socioeconomic factors and geographical location. The impact of vulnerability is amplified in disadvantaged communities, where low dietary diversity may coexist with food insecurity, limited access to health care, and unfavorable conditions for childcare [7]. These challenges have been more pronounced in rural and eastern regions of Indonesia, where socioeconomic disadvantage and limited access to diverse foods may contribute to lower dietary diversity among young children [8]. Recent food systems-focused analyses have underscored the importance of food affordability and accessibility as significant factors influencing household capacity to provide diverse complementary feeding options [42]. This supports the assertion that dietary diversity should be considered beyond the personal practice of infant feeding and instead as an outcome shaped by interacting determinants at the household, community, and food system levels.

### 4.5. Programmatic and Policy Implications

The evidence synthesized in this review suggests gaps may remain between national stunting prevention strategies and complementary feeding practices at the household level. While nutrition strategies in Indonesia focus on the first 1000 days, the implementation of dietary diversity counseling may not always be sufficiently tailored to local food environments and household circumstances [14,15]. Community-based programs such as Posyandu provide relevant platforms for applying dietary diversity concepts and offering food-based recommendations focused on affordable, acceptable foods in communities [43,44]. Growing evidence suggests that maternal nutrition literacy and nutritional knowledge are associated with child nutritional outcomes during early childhood [34].

The critical role of healthcare professionals in ensuring the effective implementation of national stunting prevention policies at a household dietary behavior change level has gained increasing attention. Evidence from an Indonesian case study highlights the potential role of healthcare professionals in stunting prevention and suggests enhancing their capacity in order to provide context-specific nutrition and dietary diversity advice may be relevant for supporting complementary feeding practices [43]. Beyond the capacity of single health professionals, the interprofessional collaboration (IPC) model has been proposed as a potential approach to addressing fragmented health services of stunting management in Indonesia. A systematic review of IPC guidelines for managing stunting reported potential improvements in the delivery of stunting interventions when a multidisciplinary team approach involving medical doctors, nutrition experts, midwives, and community health workers is implemented to manage stunting cases within communities [45]. The implementation of the IPC framework has been operationalized in Indonesia’s Puskesmas setting, whereby an expert agreement suggested that structured IPC processes could support the effectiveness of stunting detection, referral, and dietary counseling of young children [46]. In addition to facility-based healthcare workers, Bidan Desa (village midwives) may help reach pregnant women, new mothers, and young children at the community level, serving as a primary point of contact for health and nutrition information in rural and underserved areas [44,47]. These findings provide a basis for considering stronger integration of dietary diversity counseling within existing community and primary healthcare platforms, although the effectiveness of specific implementation models should be evaluated in local settings.

Within this policy context, dietary diversity may be considered one relevant component of Indonesia’s broader nutrition agenda, including institutional feeding initiatives such as Makan Bergizi Gratis (MBG) program. While the MBG initiative covers multiple target groups, its emphasis on improving access to nutritious foods is relevant to the broader goal of improving dietary quality and preventing malnutrition among children [15]. However, the reviewed studies did not directly evaluate the effect of MBG on dietary diversity or nutritional status. Therefore, the relevance of dietary diversity principles to MBG should be regarded as a policy implication of the evidence rather than as an established effect of the program. More broadly, evidence on complementary feeding and nutrition interventions suggests that feeding programs should consider not only the provision of adequate calories but also the diversity of foods provided, particularly animal source foods and fruit and vegetable portions [13,31,39]. Additionally, household food environments and caregivers’ food preferences have been associated with children’s consumption of diverse complementary foods, suggesting that household nutrition education should accompany broader food-based interventions [9,40]. Aligning institutional feeding recommendations with complementary feeding principles may therefore provide an opportunity to reinforce dietary diversity messages across different nutrition settings, although the effectiveness of such alignment requires further evaluation.

Overall, this review offers an insight into the potential biological pathways and context-specific factors that may help explain the relationship between dietary diversity and child growth. By integrating these potential pathways, socioeconomic determinants, and policy perspectives, this review provides a more comprehensive understanding of the relationship between dietary diversity and child growth in the Indonesian context. Given the predominantly observational nature of the evidence, dietary diversity should be considered one component of broader strategies to address child undernutrition rather than as a standalone intervention. The available evidence supports consideration of dietary diversity as an intervention-relevant component of efforts to address infant and child undernutrition in Indonesia, while also highlighting the importance of addressing the biological, socioeconomic, household, and policy context in which dietary practices occur.

### 4.6. Actionable Recommendation Strategies

Based on the evidence synthesized in this review, several potential areas for intervention and future research may be considered to support complementary feeding practices and thereby increase dietary diversity. At the household and community levels, Posyandu cadres could be supported with dietary counseling materials targeting the promotion of affordable and locally available animal-source foods (ASFs), such as eggs, chicken liver, tempeh-tofu, and small fish, as potential components of complementary feeding for children aged 6–23 months. Such counseling may also address cultural food beliefs, food taboos, affordability, and accessibility barriers. At the healthcare facility level, Puskesmas could consider structured interprofessional collaborative approaches involving doctors, nutritionists, midwives (including Bidan Desa as community-level points of contact), and community health workers to support the identification of young children and their parents who may require referral, follow-up, or dietary counseling. At the program level, the Makan Bergizi Gratis (MBG) program could consider incorporating dietary diversity principles into relevant food provision and nutrition education activities, including attention to ASF and foods contributing iron and vitamin A. However, the specific effectiveness of these approaches for improving dietary diversity and nutritional status requires further evaluation.

At the policy and research level, longitudinal and implementation-focused studies are needed to evaluate the effectiveness of maternal nutrition literacy programs, complementary feeding counseling, food-based interventions, and local food system strategies in improving dietary diversity and nutritional outcomes in Indonesian children. Further research may also examine potential biological pathways, including micronutrient adequacy and gut health, to clarify mechanisms underlying the observed associations. Such evidence could help inform context-specific strategies for strengthening complementary feeding practices in Indonesia.

## 5. Conclusions

This review suggests that dietary diversity during complementary feeding may be associated with more favorable anthropometric outcomes among children aged 6–23 months, although the strength and consistency of these associations varied across studies. The potential biological pathways discussed, including micronutrient adequacy and gut microbiome health, represent plausible explanations for these associations but were not directly established as mediating pathways in the included studies. In the Indonesian context, these associations occur within a broader landscape of socioeconomic inequalities, maternal nutrition literacy, cultural feeding barriers, and food system constraints that may limit access to diverse, nutrient-dense foods, particularly animal-source foods. Given the predominantly observational nature of the available evidence, dietary diversity should be considered one component of broader strategies to support child nutritional health rather than as a standalone determinant of nutritional status. Addressing child undernutrition in Indonesia may benefit from integrated strategies that consider dietary quality, caregiver behavior change, and health system strengthening within the broader first 1000 days framework, while recognizing that this review specifically focused on children aged 6–23 months.

## Figures and Tables

**Table 1:** Characteristics of studies included in the primary evidence synthesis.

Author (Year)	Setting; Design; Sample	Dietary Diversity/CF Measure	Nutritional Outcome	Key Findings
Onyango et al. (2014) [16]	21 countries; DHS secondary analysis; 6–23 mo	MDD, MMF, and MAD based on IYCF indicators	LAZ	MDD was associated with LAZ in 12 of 21 countries in adjusted analyses, although the magnitude and direction of the association varied across countries and age groups.
Paramashanti et al. (2017) [17]	Bantul, Yogyakarta, Indonesia; cross-sectional; *n* = 189	MDD ≥ 4 of 7 food groups	Stunting	Poor dietary diversity (<4 food groups) was strongly associated with stunting in multivariable analysis (OR = 16.76, 95% CI: 6.77–41.51). Household economic status was also considered an effect modifier in the association between dietary diversity and stunting.
Diana et al. (2017) [18]	Sumedang, West Java, Indonesia; prospective cohort; *n* = 230	WHO CF indicators, dietary diversity, MDD ≥ 4 of 7 food groups	LAZ, WAZ, WLZ	MDD was not significantly associated with LAZ, WLZ, or WAZ at 12 months. Iron-rich/iron fortified infant food consumption was associated with higher LAZ (β = 0.22, 95% CI: 0.01–0.44), while fortified infant food consumption was associated with higher LAZ (β = 0.29, 95% CI: 0.09–0.48) and WAZ (β = 0.14, 95% CI: 0.02–0.26) after adjustment.
Ahmad et al. (2018) [19]	Aceh, Indonesia; cross-sectional; *n* = 392	MDD (mean of 3 repeated 24 h recalls) and other IYCF indicators; MDD ≥ 4 of 7 food groups	Stunting, wasting, underweight	MDD was not significantly associated with wasting, underweight, or stunting in bivariate analyses (*p* = 0.667, 0.686, and 0.816, respectively). Fortified food consumption was significantly associated with stunting in bivariate analysis (*p* = 0.015), although no effect estimate was reported.
Komaruddin et al. (2019) [20]	East Jakarta, Indonesia; cross-sectional; *n* = 120	Dietary diversity based on food-group consumption, MDD ≥ 4 of 7 food groups	Stunting	Poor dietary diversity was strongly associated with stunting in the multivariate analysis (OR = 6.76). Household economic status was considered as a potential effect modifier of this association.
Nai and Renyoet (2020) [21]	Yogyakarta, Indonesia; cross-sectional; *n* = 135	Dietary diversity/MDD ≥ 4 of 7 food groups	Stunting	Poor dietary diversity was significantly associated with stunting after adjustment (OR = 2.91, 95% CI: 1.21–7.02; *p* = 0.017)
Anin et al. (2020) [22]	Northern Ghana; cross-sectional; *n* = 581	MDD, MMF, MAD and other IYCF indicators	Stunting, wasting	MDD was significantly associated with stunting in bivariate analysis (*p* < 0.001), but was not retained in the final multivariable model, indicating that the association was not independent after adjustment. Iron-rich food intake was associated with stunting (AOR = 0.48, 95% CI: 0.25–0.91).
Aboagye et al. (2021) [23]	32 countries in Sub-Saharan Africa; DHS secondary analysis; *n* = 48,968	MDD	Stunting, wasting, underweight	Adequate MDD was associated with lower odds of stunting (aOR = 0.88, 95% CI: 0.83–0.94), underweight (aOR = 0.83, 95% CI: 0.77–0.91), and wasting (aOR = 0.87, 95% CI: 0.78–0.97) in the fully adjusted model.
Samosir et al. (2023) [24]	Indonesia; secondary analysis of Riskesdas 2018; *n* = 14,216	Food consumption diversity/MDD	Nutritional status: normal, stunted, severely stunted	Greater food consumption diversity was associated with higher odds of a more favorable nutritional status category in the adjusted ordinal logistic regression model (OR = 1.15, 95% CI: 1.07–1.24; *p* < 0.001).
Paramashanti et al. (2024) [25]	Bantul, Indonesia; cross-sectional; *n* = 189	MDD based on ≥4 of 7 food groups	Concurrent stunting and overweight (CSO)	Meeting MDD was associated with lower estimated odds of CSO in the adjusted model (aOR = 0.14, 95% CI: 0.03–2.43), although the association was not statistically significant.
Tan et al. (2024) [26]	Vietnam; national survey secondary analysis	MDD ≥ 5 of 8 food groups and MAD	Stunting, underweight, overweight, micronutrient deficiencies	Adequate MDD was associated with lower odds of stunting (aOR = 0.61, 95% CI: 0.41–0.92), iron deficiency (aOR = 0.69, 95% CI: 0.54–0.88), and iron deficiency anemia (aOR = 0.63, 95% CI: 0.46–0.88) in adjusted models.
Lestari et al. (2024) [27]	Bantul, Indonesia; cross-sectional; *n* = 167	IDDS based on ≥5 of 8 food groups	Stunting	Poor dietary diversity was significantly associated with stunting (OR = 2.56, 95% CI: 1.42–4.14; *p* = 0.005).
Surmita et al. (2025) [28]	West Java, Indonesia; SSGI 2022 secondary analysis; *n* = 6700	MDD based on ≥5 of 8 food groups	Stunting	Inadequate MDD was significantly associated with higher odds of stunting in the primary multivariable model (aOR = 1.52, 95% CI: 1.27–1.82; *p* < 0.001), and the association remained significant after further adjustment for additional socioeconomic and maternal factors.
Ariftiyana et al. (2025) [29]	Bantul, Yogyakarta, Indonesia	MDD, MMF, MAD, timely introduction of CF, and ASF consumption	Stunting	Inadequate MDD was significantly associated with stunting after multivariable adjustment (AOR 5.17, 95% CI: 1.57–16.99). Other complementary feeding indicators, including MMF, MAD, timely introduction of CF, and ASF consumption, were not significantly associated with stunting after adjustment.
Moshi et al. (2025) [30]	Arusha, Tanzania; community cross-sectional; *n* = 279	MDD, MMF, MAD, and food-group consumption	Stunting, wasting, underweight, anemia	MDD was not significantly associated with underweight, wasting, stunting, or anemia after adjustment. Consumption of flesh foods was associated with lower odds of underweight (AOR 0.16, 95% CI: 0.04–0.73) and anemia (AOR 0.16, 95% CI: 0.07–0.41), while dairy consumption was associated with higher odds of anemia (AOR 10.24, 95% CI: 2.79–37.57).

aOR, adjusted odds ratio; ASF, animal-source foods; CF, complementary feeding; CI, confidence interval; CSO, concurrent stunting and overweight; DHS, Demographic and Health Survey; IDDS, individual dietary diversity score; IYCF, infant and young child feeding; LAZ, length-for-age z-score; MAD, minimum acceptable diet; MDD, minimum dietary diversity; MMF, minimum meal frequency; OR, odds ratio; SSGI, Indonesian Nutritional Status Survey (Survei Status Gizi Indonesia); WAZ, weight-for-age z-score; WHO, World Health Organization; WLZ, weight-for-length z-score. MDD Definitions reflect the criteria used in the original studies. Earlier studies used the previous threshold of ≥4 of 7 food groups, whereas studies applying the updated WHO/UNICEF indicator used ≥5 of 8 food groups [6].

## Data Availability

No new data were created or analyzed in this study. Data sharing is not applicable to this article.

## Abbreviations

The following abbreviations are used in this manuscript:

ASF	Animal-Source Foods
CF	Complementary Feeding
DDS	Dietary Diversity Score
DHS	Demographic and Health Survey
IGF-1	Insulin-like Growth Factor-1
IPC	Interprofessional Collaboration
IYCF	Infant and Young Child Feeding
LAZ	Length-for-Age Z-score
MBG	Makan Bergizi Gratis (Free Nutritious Meal Program)
MDD	Minimum Dietary Diversity
Posyandu	Pos Pelayanan Terpadu (Integrated Health Service Post)
Puskesmas	Pusat Kesehatan Masyarakat (Community Health Center)
Riskesdas	Riset Kesehatan Dasar (Basic Health Research)
SSGI	Survei Status Gizi Indonesia (Indonesian Nutritional Status Survey)
UNICEF	United Nations Children’s Fund
WAZ	Weight-for-Age Z-score
WHO	World Health Organization
WHZ	Weight-for-Height Z-score
